# Secure Ileal Pouch–Anal Anastomosis for Histologic Indeterminate Colitis

**DOI:** 10.3390/jcm14238390

**Published:** 2025-11-26

**Authors:** Amosy E. M’Koma

**Affiliations:** 1 Department of Biochemistry, Cancer Biology, Neuroscience and Pharmacology, Meharry Medical College School of Medicine, Nashville, TN 37208-3500, USA; amkoma@mmc.edu or amosy.e.mkoma@vumc.org; 2 Department of Pathology, Anatomy and Cell Biology, Meharry Medical College School of Medicine, Nashville General Hospital, Nashville, TN 37208-3599, USA; 3 Division of General Surgery, Section of Colon and Rectal Surgery, Vanderbilt University School of Medicine, Nashville, TN 37232-0260, USA; 4 The American Society of Colon and Rectal Surgeons (ASCRS), 2549 Waukegan Road, #210, Bannockburn, IL 600015, USA; 5 International Society of University Colon and Rectal Surgeons, 109 Partin Street, Chapel Hill, NC 27514, USA

**Keywords:** inflammatory bowel disease, indeterminate colitis, Crohn’s colitis, ulcerative colitis, *de novo* Crohn’s disease, DEFA5, colectomy, surgical procedure, proctocolectomy, ileal pouch–anal anastomosis, diagnosis, diagnosis ambiguity, clone 18A, clone 4F5, colonic ileal metaplasia, colonoscopy, endoscopy

## Abstract

**Background/Objectives:** Indeterminate colitis (IC) is an erroneous diagnosis for predominantly colonic inflammatory bowel disease (IBD) when there is a non-definitive foundation of the benchmark for ulcerative colitis (UC) and Crohn’s colitis (CC) after a combined state-of-the-art classification system of clinical, endoscopic, radiologic, and histologic tools are used. This confounds an effective surgical regimen; specifically pouch surgery, “the restorative proctocolectomy with ileal pouch–anal anastomosis (PRC-IPAA)”. Transforming the distinction between UC and CC in otherwise IC into authentic UC and CC requires priority attention when considering a patient’s candidacy for RPC-IPAA. RPC-IPAA is the accepted standard curative surgical procedure in the treatment for UC (and Familial Adenomatous Polyposis (FAP)). Further, inapproximate/incorrect diagnosis and treatment can sustain potential long-term morbidity from inaccurate and unnecessary surgery and cost. **Methods:** In trying to resolve these diagnostic ambiguities, the current study advances our understanding by showing the expression of human alpha defensin 5 (DEFA5 alias HD5) restricted in the colon crypt mucosal lining areas, and by identifying the cells of the small intestine (ileum) “colonic ileal metaplasia” in CC that may serve as a biomarker to portray/ascertain authentic CC and UC among IC cohorts, with a positive predictive value (PPV) of 96 percent. **Results**: Hence, the imprecise diagnosis of IC largely would be circumvented. This new diagnostic tool offers instant tangible benefits over existing diagnostic pathways. The journey toward its widespread clinical use is now subject to logistical and regulatory defiance, which all emerging molecular diagnostic technologies inevitably encounter. **Conclusions**: The aim of this communication is to provide a summary of the currently available diagnostic advances relating to surgical management for IC in clinical settings, and the related challenges. Further, I briefly discuss aspects of its pathophysiology, surveillance, and diagnostic assay development.

## 1. Introduction

Predominantly colonic inflammatory bowel disease (IBD) encompasses ulcerative colitis (UC) and Crohn’s colitis (CC), which are characterized by chronic, relapsing inflammation of the colon and rectum; involvement of the anus and perianal region is a feature specific to Crohn’s disease. Studies at Meharry Medical College, the Vanderbilt University Medical Center [1], and other institutions [2,3,4] found an estimated 15% of patients with colonic IBD to have an inaccurate diagnosis of “indeterminate colitis (IC)”, which occurs when the diagnostic classification features for UC and CC are inconclusive after a comprehensive evaluation including clinical, endoscopic, radiological, and histological examinations. The dedifferentiation of IBD often leads to unnecessary and ineffective interventional regimens, subsequent morbidity, and cost. The prevalence of IC is about 22 persons per 100,000 people, and it is commonly observed with a high incidence in female Caucasians [3]. A total proctocolectomy (TPC) or restorative proctocolectomy with ileal pouch–anal anastomosis (RPC-IPAA) is the surgical treatment option for UC patients who become refractory to pharmaceuticals [5,6,7]. Further, TPC and/or RPC-IPAA is carefully indicated for select patients with CC [8,9], and for patients with hereditary nonpolyposis colorectal cancer or synchronous colon cancers [10,11]. Furthermore, for patients with Familial Adenomatous Polyposis (FAP), a prophylactic TPC is indicated because they possess the hereditary adenomatous polyposis coli gene and inevitably develop colorectal cancer in their lifetimes [12]. Accurate diagnosis in patients presenting with colonic IBD and their mimics is consequentially essential, and it is important for a tailored surgical intervention, because each entity may require a specific approach or regimen, the delivery of which may have broad implications.

The RPC-IPAA procedure requires the replacement of the resected entire colon and rectum by a pouch formed from the terminal/distal ileum and sutured to the anal canal, above the dentine/pectinate/mucocutaneous line, a significant crucial anatomical landmark in the anal canal that separates the upper and lower parts, preserving the anal sphincters and maintaining numerous physiological and clinical features [13,14]. Briefly, the RPC-IPAA reconstruction gives the gastrointestinal tract luminal continuity, defecation, deferral, discrimination, and fertility. Early observations reported complication rates of over 50 percent and failure rates as high as 35 percent [15,16]. These observations prompted the importance of patient selection, and only patients with UC and FAP were recommended candidates for RPC-IPAA surgery [17,18]. Crohn’s colitis was contraindicated [17,18]. Over the years, however, improved diagnostic tools, technical modifications, and surgical expertise have been developed so that the procedure can now be performed safely with a low complication and failure rates of less than five percent [19,20,21]. Hence, indications for the intervention have widened, especially for older patients and those with a history of perianal disease, who can be selectively chosen as candidates or not for the RPC-IPAA procedure [22]. Intriguingly, some IBD Centers advocate for performing RPC-IPAA surgery in patients with CC [18,23]. However, though RPC-IPAA surgery is feasible with indifferent functional outcomes between CC and UC, even in highly selected patients with known preoperative CC or a proctocolectomy pathology, pouch failure rates stay higher than in patients with UC [9]. Therefore, it is not surprising that IC is not a contraindication for pouch surgery at many IBD Centers worldwide [22,23]. However, subsequent morbidity necessitating excision of the pouch is reported in 20 to 30 percent of patients [22,23]. These patients will most likely experience complications such as intra-abdominal and perineal sepsis and fistulas, and they are likely to require several operations, including one to excise their pouch, losing approximately 50 cm of the small bowel. Mortality from their complications is unlikely, and it is also unlikely that they will have subsequent short bowel syndrome (SBS) [24,25,26]. Conversely, approximately 70 percent to 80 percent of patients will have acceptably functional pouches, despite the fact that some of them may require further surgery due to adverse events [27]. The alternative to RPC-IPAA is total PTC and permanent terminal ileostomy [23,25,28].

In trying to resolve unmet diagnostic delay and ambiguity gapes in colonic IBD, the current study advances our understanding by showing the expression of human alpha defensin 5 (DEFA5 alias HD5) restricted in the colon crypt mucosal lining areas, and it identifies the cells of the small intestine (ileum) “colonic ileal metaplasia” in CC that may serve as a biomarker to characterize/delineate and ascertain authenticity for CC and UC among the IC cohorts, with a positive predictive value (PPV) of 96 percent [1]. Hence, the imprecise diagnosis of IC largely would be circumvented. This new diagnostic tool offers instant tangible benefits over existing diagnostic pathways. The journey toward its widespread clinical use is now subject to logistical and regulatory defiance, which all emerging molecular diagnostic technologies inevitably must withstand [5]. The aim of this communication is to provide a summary of the currently available diagnostic advances and surgical management for IC, and the challenges in clinical settings. Further, I briefly discuss aspects of its pathophysiology, surveillance, and diagnostic assay development advances.

## 2. Methods

A search of the diagnostic literature and treatment recommendation guidelines for IBD and IC was performed using predetermined protocols. The search used general engines like Google and specific databases including the Cochrane Database, MEDLINE, EMBASE, PubMed, and the Cumulative Index of Nursing and Allied Health Literature (CINAHL), covering the period between January 1980 and June 2025. The process also included a review of guidelines from IBD-associated society organizations and official agencies, i.e., the American Gastroenterological Association (AGA), the American Society for Gastrointestinal Endoscopy (ASGE), the British Society of Gastroenterology (BSG), the International Foundation for Gastrointestinal Disorders (IFGD), the American Society of Colon and Rectal Surgeons (ASCRS), the American College of Gastroenterology (ACG), the Society of American Gastrointestinal and Endoscopic Surgeons (SAGES), the International Organization for the Study of Inflammatory Bowel Disease (IOIBD), the World Health Organization (WHO), the U.S. Food and Drug Administration (USFDA), the European Medicines Agency (EMA), European Crohn’s and Colitis (ECC), American Crohn’s and Colitis (CCFA), and the Canadian Association of Gastroenterology (CAG). The quality of reporting followed guidelines for meta-analyses of observational studies (MOOSE).

### Ethics

This communication was approved by the Meharry Medical College (IRB file numbers: 100916AM206 (date of 16 September 2010), 18-08-851 (date of 12 September 2018), 22-08-1235 (date of 15 August 2022), and 22-08-1244 (date of 26 August 2022) and Vanderbilt University (IRB file numbers: 080898 (date of 29 September 2008) and 100581 (date of 1 June 2010). Institutional Ethical Review Boards ensured this study was conducted in accordance with the World Medical Association (WMA) Declaration of Helsinki – Ethical Principles for Medical Research Involving Human Participants. Patient samples for the included studies were obtained from the NIH-funded Digestive Disease Research Center (PI: David A. Schwartz, MD), Vanderbilt Gastrointestinal Biospecimen Repository, and the Cooperative Human Tissue Network at Vanderbilt University Medical Center (VUMC) (PI: Mary K. Washington, MD, PhD), in collaboration with the Meharry Medical College Human Tissue Acquisition Shared Resource Core (PI: Billy R. Ballard, DDS, MD). The availability of a detailed IBD patient database registry at VUMC made a chart review and follow-up surveillance possible. Medical record data on patient demographics, preoperative variables prior to and after RPC-IPAA surgery, surveillance of endoscopic and clinical findings, and medical and surgical treatment history were retrieved retrospectively. Patient informed consent was given, and participation in this study was voluntary.

## 3. Indeterminate Colitis

**Figure 1:**
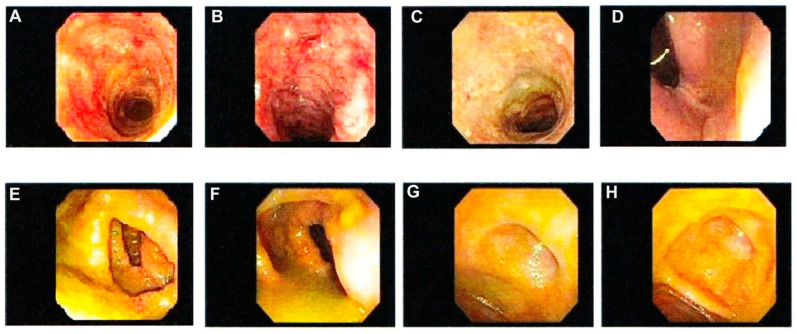
Depicts indeterminate colitis (IC) endoscopy images. IC is diagnosed when transmural lympoid aggregates are present. Inconsistence expiriences have been reported in reviewing the cases of IC. In some reports the disease is most likely to behave clinically like UC [51,52] and much less likely to develop into obvious clinical CC [35] while others found the reverse is true [53]. IC is provisional diagnosed for IBD when transmural lymphoid aggregates are present but it is not possible to definitively classify the disease as CC or UC. (**A**) fulminant IC of the transverse colon, (**B**) fulminant IC of the descending colon, (**C**) IC of the sigmoid colon, (**D**) IC of rectum (**E**) IC of the mid-ascending colon, (**F**) IC of the ileocecal, (**G**) IC of the appendiceal orifice, (**H**) IC of the cecum.

**Figure 2:**
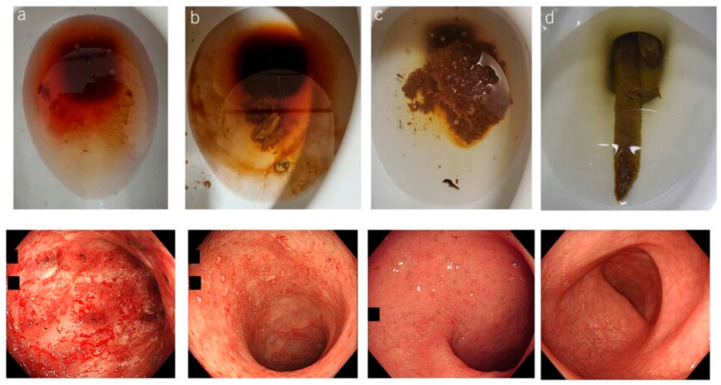
Endoscopic photographs of patients with ulcerative colitis (UC) according to their endoscopic activity. Ulcerative colitis endoscopic index of severity (UCEIS). (**a**) UCEIS 7, (**b**) UCEIS 4, (**c**) UCEIS 2 and (**d**) UCEIS 0. Reproduced with permission from Lee JW et al., Am J Gastroenterol 120(1):213-224, 2025.

**Figure 3:**
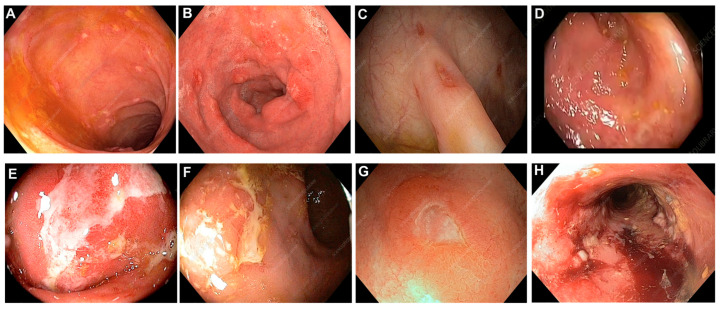
Endoscopic view of colonic Crohn’s colitis (CC). (**A**–**C**), view of colonic Crohn’s colitis (CC) with patchy inflammation, ulcers, and a cobblestone appearance of the mucosa; (**D**), CC of sigmoid colon; (**E**,**F**), CC of the Rectum; (**G**), Ulceration in the Rectum; (**H**), Rectal stricture. Reproduced with permission from GASTROLAB/SCIENCE Photo Library: Licensee—American African Health Team, LLC, https://americanafricanhealthteam.com/ (accessed on 5 August 2025).

The term “indeterminate colitis (IC)” was originally introduced in 1978 to describe surgical specimens from IBD patients undergoing colectomy, when the histological features were not characteristic of either CC disease or UC [29,30,31,32,33]. Distinguishing UC from CC is often complex and challenging [34]. In particular, in the interim uncertainty prodromal-stage cases of extensive ulceration, the two diseases may visually be characteristically indistinguishable [35,36]. Inadequate differentiation of diagnostic features of UC and CC, as illustrated in Figure 1A–H may lead to an inconclusive diagnosis of IC even when a combined state-of-the-art classification systems of clinical, endoscopic, radiological, and histological tools are used [33,37,38,39]. Up to 15% of colonic IBD cases are labeled as IC when non-definitive evaluation criteria for either UC or CC have been established from colonoscopy biopsies, or at colectomy [32,33,40,41,42,43]. Most patients with IC do eventually evolve to a definite diagnosis of CC or UC after long-term follow-up surveillance, which can indeed span many years [1,25]. The approximate percentage of patients who have their diagnosis changed from presumed definitive UC to *de novo* Crohn’s disease (CD) after RPC-IPAA is within the range cited of 15% based on the postoperative follow-up clinical observations and histopathology changes, and development of *de novo* CD in the ileal pouch [17,23,25,44]. Despite the introduction of newer cutting-edge diagnostic technology modalities, these figures have not changed for over six decades [45]. One-half of these patients will require pouch excision or diversion [23]. Much of the diagnostic uncertainty arises from the overlap of clinical and histopathological features of both conditions, making CC appear like UC and vice versa, Figure 1A–H [23,34]. The characteristics of UC result in inflammation and ulcerations confined to the mucosal lining areas and scarcely extending to submucosal layer of the colon and rectum, Figure 2a–d [45,46,47]. Meanwhile, CC differs in that inflammation may result through the intestine walls (transmural) with skip lesions, Figure 3A–H [33,45,46,47,48]. Further, CC may also involve other body organs outside boundaries of the gastrointestinal tract (GIT) system through fistulation [49,50]. The current surgical treatment recommendation options for UC and CC are still debatable, and guidelines emphasize the urgency of surgical options for patients with medically refractory UC [31]. Therefore, an accurate diagnosis is of supreme importance in terms of determining the evidence-based patient candidacy for surgical intervention and personalized preoperative counseling for the possible subsequent outcome. To date, colorectal surgeons provide accounts of the challenge, where some patients with IC may appear to have UC, and others may appear to have CC. The former may be considered candidates for RPC-IPAA, whereas the latter may be advised to undergo total PTC. Another alternative approach is to perform a subtotal colectomy with terminal ileostomy initially and then, depending on the pathology results, proceed with pouch surgery if the pathological diagnosis is UC. If the pathological diagnosis is IC, one could suggest waiting to see whether small bowel or perianal manifestations develop signs, which would suggest the diagnosis of CC (severe pouchitis, proctitis, cuffitis, strictures, abscess sinus tracks, fistulae, etc.). Secondly, if one adopts a policy of performing RPC-IPAA in patients with refractory IC, it is probably reasonable to be carefully selective in the choice of candidates. It is a wise move that only patients with IC and or CC who are young, without comorbidities, and who are signed off as psychologically stable after counseling should be considered for RPC-IPAA, because morbidity rates are higher in these patients [7,8,9].

Inconsistence experiences have been reported in reviewing the cases of IC. In some reports, the disease is most likely to behave clinically like UC [51,52] and much less likely to develop into clear clinical CC [35], while others found the reverse is true [53]. Diagnostic authentication validation studies for CC versus UC among IC cohorts using the DEFA5 bioassay test with a first-clinic-visit endoscopy biopsy are underway, https://cdn.clinicaltrials.gov/large-docs/71/NCT05663671/Prot_000.pdf (accessed date 10 August 2025).

## 4. Criteria for Making a Diagnosis

**Figure 4:**
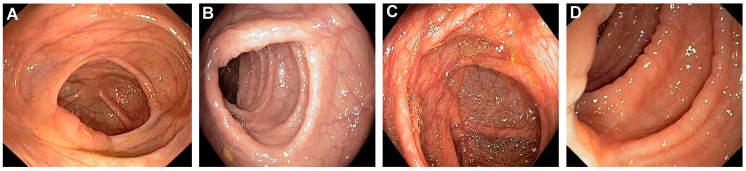
Healthy Colon. (**A**) Ileocaecal valve, (**B**) ascending colon, (**C**) hepatic flexure of the colon, (**D**) rectum. Reproduced with permission from GASTROLAB/SCIENCE Photo Library: Licensee—American African Health Team, LLC, https://americanafricanhealthteam.com/ (accessed on 5 August 2025).

Endoscopically, the normal colonic mucosa is lined with simple columnar epithelium, which lacks the villi and contains crypt of Lieberkühn, Figure 4. The criteria for determining a diagnosis of IBD require an experienced endoscopy gastroenterologist and gastrointestinal pathologist to review the histological pathology [35,54]. The gross and histological features quoted in the pathology literature as being useful in distinguishing the UC vs. CC are evaluated. These quotes include linear ulcers on gross examination, granulomas of the sarcoid type, terminal small intestines/ileal inflammation (including backwash ileitis), slit-shaped ulcers, patchy mucosal inflammation, skip zones, and transmural inflammation. These are considered to be present when lymphoid aggregates are noted in the subserosa and/or the deep submucosal zone [35,54,55]. Serosal inflammation that is not in the form of lymphoid follicles should be disregarded. This is present mainly in patients with fulminant colitis immediately beneath/below deep broad-based ulcers, Figure 2a [35,36].

## 5. Diagnostic Dilemmas for IBD

To date, there is no single diagnostic “gold standard” tool for IBD [34]. Clinicians use a comprehensive combined classification system that includes clinical, endoscopy, radiologic, and histopathology findings in order to diagnose CC and UC [45,56]. Notably, even with the combination of these diagnostic utilities, 15% to 30% of IBD patients cannot be accurately diagnosed [34,57,58]. This pitfall has a significant implication when determining a patient’s candidacy for the RPC-IPAA intervention [17,34]. Curative treatment for UC recommends RPC-IPAA [17,19,44,59,60,61,62]. The successfulness of RPC-IPAA is largely dependent on careful patient selection combined with a conscientious surgical technique and diagnosis precision [19,57,63,64]. Currently available clinical experiences suggest that it is painstaking and strenuous to identify patients with CC who are likely to have a successful outcome after RPC-IPAA surgery [17,44,57,63]. Thus, CC should remain carefully indicated for pouch surgery but is an acceptable treatment option for patients with UC and for those IC patients who are predicted to develop UC [61,65,66,67,68,69]. These findings are beyond reasonable doubt informative in notifying the role of RPC-IPAA in preoperative counseling and decision-making with candidate patients.

## 6. Reclassification of Indeterminate Colitis to Definitive Ulcerative Colitis or Crohn’s Colitis

Under the umbrella of predominantly colonic IBD, it is pivotal to delineate authentic CC and UC amongst IC cohorts for informed pouch surgery decisions and outcomes [1,53]. Several attempts to develop a molecular “gold-standard diagnostic tool” to identify a reliable bioassay for delineating IC into authentic CC and UC have been ongoing for decades [34,45,70,71,72,73,74,75,76]. Recently, a discriminative breakthrough was made with the discovery of aberrant expression of DEFA5 in colonic tissues linked to the distinct pathogenesis of authentic CC [1,53]. To break down patients with IC into actual UC and CC is of primary importance for RPC-IPAA decision-making [17,19,44,59,60,77,78,79]. The success of RPC-IPAA surgery depends largely on the “correct diagnosis” as UC (or FAP) [19,57,63,64]. Observational clinical experiences suggest that patients with CC have higher pouch failure outcomes subsequent to RPC-IPAA surgery compared to UC [8,9,17,44,57,61,63,65,66,67,68,69].

### 6.1. DEFA5 Is a Promising Diagnostic Biomarker

Among potential diagnostic biomarkers, human alpha defensin 5 (DEFA5 alias HD5) has emerged as a promising candidate due to its differential expression patterns in CC and UC, making it a valuable target for IBD-subtype diagnostic development [1,80]. In colonic mucosal crypt layers, DEFA5 is ectopically expressed in patients with CC and IC cohorts that are foreseen as CC, and it is demonstrated that this expression pattern holds high diagnostic potential in distinguishing CC versus UC pathologies amongst IC patient cohorts [1,53]. DEFA5 expression is markedly exalted 118-fold in CC when compared with UC, whereas its levels in UC are trace, distinguishing CC from UC with high specificity [1,53,80]. These differential expression patterns underscore the potential capability of DEFA5 as a biomarker for IBD-subtype classification execution.

### 6.2. Advances in Assay Development for IBD Diagnostics

Significant advancements are being made through the utilization of the DEFA5 biosignature, which provides precision in resolving the diagnostic ambiguity of IC into authentic CC and UC. High levels of DEFA5 are reliably linked to a diagnosis of CC among IC cohort patients to accurately classify patients who might otherwise remain without a definitive diagnosis for years [1,53,80]. These innovative inventions are protected by three granted patents, US 11427852 B2, US 12174200 B2, and US 12281351 B2, and four International patents/applications WO 2018/175913 A1 (WIPO), WO 2018/237064 A1 (WIPO), EP3602041A1/A4 and CA-3056911-A1 exclusively assigned to Meharry Medical College in the realm of IBD diagnosis biomarkers, which could change the diagnostic landscape and lend mechanistic insights into IBD pathogenesis, allowing us to move beyond traditional methods, and thus paving the way for more tailored therapies and improved patient outcomes [80].

### 6.3. Anti-DEFA5 Monoclonal Antibodies

Two established tested functional novel anti-DEFA5 monoclonal antibodies (mAbs), clones 1A8 and 4F5, are validated for specificity, selectivity, and cross-reactivity in recognizing the endogenous and recombinant DEFA5 protein [80]. Biotechnology development studies on mAbs, clones 1A8 and 4F5, in larger IBD cohorts are underway to establish efficacy and safety (Trial NCT05663671).

## 7. Transcriptome Studies

Quantitative global expression profiles of RNA levels, generated using an oligonucleotide microarray and genome-wide transcriptome analysis, were investigated to identify transcriptional signatures present in colonic biopsy tissues obtained from UC and CC mucosa and submucosal linings [71,74]. The genomic patterns noted show greater intensity in CC as compared to UC, indicative of a greater degree or different type of inflammation response in the tissues’ underlying layers [75], and these may serve as a resource for professionals involved in gene expression data mining in a variety of clinical settings, particularly for the differential diagnosis of UC and CC in IC.

## 8. Proteomic Profiling

Studies have developed a proteomic technology, Matrix-Assisted Laser Desorption/Ionization Mass Spectrometry (MALDI-MS) technology, for proteomic profiling of histologic mucosal and submucosal tissue layers for analyses along with bioinformatics technologies to delineating UC and CC [70,75]. These studies identified and compared protein profiles, which had the necessary (i) specificity, (ii) sensitivity, (iii) discrimination, and (iv) predictive capacity to determine the heterogeneity of IBD subtypes and the ability to delineate UC and CC molecularly [70,75]. These rare biometrics based on the lining of the colonic pathology are ectopically independent of the tissue of origin and the characteristics of the ileum, consistent with “colonic ileal metaplasia” [53], representing disease-specific inflammatory markers [70,75].

## 9. Blood-Based Biomarkers

In contrast to colon surgical pathology tissue resections, peripheral blood is a much more accessible source of cells that might be used to distinguish between CC and UC. Circulating peripheral blood cytokines (variables) are responsible for signs of disease surveillance. Cytokines are therefore surrogates for disease-induced gene expressions as biomarkers of the disease status [81,82]. In pursuit of this, studies on the different serum cytokine behaviors between UC and CC patients aimed to drive the development of an assay that could offer an easy, accurate, affordable, noninvasive, and fast screening test [76]. Certain cytokines were found to differ between IBD subtypes and controls [76]. A univariate analysis showed a statistically significant surge of eotaxin, GRO, and TNF-α in UC cohorts compared to controls (Ctrl); interferon γ, interleukin (IL)-6, and IL-7 in the CC group versus the Ctrl; and IL-8 in both UC and CC as compared to the Ctrl. No cytokine, chemokine, and growth factor could clearly distinguish UC from CC [76]. An analysis of the literature has shown that although several attempts have been made to define the serum cytokines, chemokine, and growth factor profiles in IBD, the results do not indicate the possibility of finding UC- versus CC-delineating biomarker(s) in the serum at this time. Despite the increased use of innovative mordent technologies, there is no single straightforward explanation for the heterogeneous results, and currently available approaches still require validation, along with confirmation on patient outcomes in large-scale clinical cohorts. Most available published reports have variably presented serum biomarkers for follow-up monitoring of the disease responses to prescribed pharmaceuticals for prognostic indicator purposes, but not for distinguishing between CC and UC or IC breakdown into authentic IBD subtypes, UC or CC [83,84,85,86,87,88,89,90,91,92,93]. Serological studies are underway, validating the development of diagnostic assays using novel anti-DEFA5 monoclonal antibodies (mAbs), clones 1A8 and 4F5 [80].

## 10. Discussion

The distinguishable clinical and histological features of CC and UC have previously been described thoroughly [94,95,96,97,98,99]. Additionally, in the 1980s, the Research Committee of the World Organization of Gastroenterology proposed a scoring system for the diagnosis of IBD and reported a diagnostic accuracy rate of 97 percent [96]. Despite that, to date, differentiating between CC and UC is still a challenge because of convergent pathologic characteristics [35,97,98,99,100,101]. Up to 15 percent of colectomy specimens excised for IBD fall into the zone “IC” [1,41,42,45]. If the diagnosis is IC, some institutions indicate the option of RPC-IPAA to pharmaceutical refractory patients [24,52,55,102]. In these patients, the risk of pouch dysfunction, or of having to have the pouch removed with subsequent permanent terminal ileostomy, was higher if the diagnosis was IC [17,103,104,105,106]. Therefore, performing an RPC-IPAA in a patient with IC is not nearly as comforting as performing it in patients with a diagnosis of definitive UC [25]. The need for IC classification into UC or CC is important for proper surgical management and care in patients suffering from IBD [107]. Patients with IC are mostly younger at diagnosis [108,109,110,111]; symptoms often begin at the age of 18 years or shortly after, with an identical gender disposition [97,111,112,113,114,115,116,117]. The reverse is true in UC, where there is a male dominance and a mean age at onset of 36–39 years [32,97,117,118,119]. These figures have not changed over six decades despite the introduction of newer cutting-edge diagnostic technologies [30,108,113,116,120,121]. A substantial number of patients with IC still show an unchanged diagnosis even after a follow-up of over 10 to 14 years of surveillance, with significant patient suffering in the interim [1,25,100,108,121]. The continued presence of an IC diagnosis over a long period of time partly supports the idea that IBD may represent a spectrum of diseases rather than just two entities, CC and UC [34,108]; beyond those, there may be other new unknown IBD subtypes or other mimic pathologies. Given the importance of the inexact IC diagnosis, it is critical that these patients be studied in a larger cohort to fully address whether DEFA5 may be used as a diagnostic biosignature to differentiate IC into true CC or UC or other forms of colitis with different pathological characteristics [1,53].

Patients with UC or some IC operated on with RPC-IPAA experience restored gut continuity, defecation, deferral, and discrimination [57,63,107,122,123,124,125,126]. However, CC patients are mistakenly diagnosed as UC or IC [30,35]. Thus, the differentiation between CC and UC has important therapeutic and prognostic implications. The currently available data show that 15% of IBD patients who undergo RPC-IPAA surgery for presumed definitive UC (or IC likely to develop UC) subsequently have the initial diagnosis changed to *de novo* CD in the ileal pouch [44,127]. The clinical experience suggests that identifying patients with CC and positive outcomes after RPC-IPAA surgery is arduous [4,44,127]. Thus, RPC-IPAA is relatively contraindicated for CC patients [4,128,129], whereas it is the standard acceptable procedure for patients with UC (and IC who are predicted likely to be UC). The reason behind this is that, inevitably, adverse pouch outcomes are significantly greater in patients with CC (+/−64%) and IC (+/−43%) compared to patients with UC (+/−22%) (*p* < 0.05) [23,41,42]. This diagnostic dilemma, along with the potential morbidity from a wrong diagnosis, unnecessary and/or inappropriate surgical indications, and associated costs, underscores the importance of a research strategy focused on improving the diagnostic accuracy of IBD using molecular biometrics [17,70,71,72,73,74,75,103,104,105,106,129]. The therapeutic management strategy may vary between these two pathologies, as does the prognosis. Early and accurate diagnosis and sub-classification of UC and CC is therefore the cornerstone for evidence-based, appropriate, personalized surgical interventional care [40,41,130]. This communication has discussed the long-term outcomes of RPC-IPAA surgery in patients with IC and the available strategies for the development of molecular bioassay tools that would help clinicians more accurately differentiate IC as being either authentic UC or CC, to guide appropriate treatment in clinical settings.

## 11. Conclusions

The DEFA5 bioassay is a reliable delineative diagnostic tool in colonic IBD, particularly for differentiating between CC and UC and in resolving ambiguous cases of IC into authentic CC and UC or a mimic colitis with different pathological characteristics. In patients with IC undergoing RPC-IPAA, those who do not develop *de novo* CD experience long-term complication-free outcomes identical to patients operated on for authentic UC, and they have 85% acceptable functioning pouches for life after operation. However, subsequent *de novo* CD of the ileal pouch following RPC-IPAA, whether it eventually develops for initially a UC or IC diagnosis, is associated with poor long-term functional outcomes. A new serological and tissue-based DEFA5 bioassay test shows the promise to change the IBD diagnostic landscape with accuracy (USPTO, US 11427852 B2, US 12174200 B2, and US 12281351 B2). Indeterminate colitis is therefore likely to be overcome.

## 12. Conference Presentations

This work was presented in part at the International Colorectal Research Summit (iCRS 2025) 29–31 August 2025 COEX Magok, Seoul, Korea; at the 2023/2024 Vanderbilt Digestive Diseases Research Center (VDDRC) Retreat, Vanderbilt University Medical Center, Nashville, TN, USA, 2 April 2024; at the 11th Dilemmas and Debates in Colorectal Surgery Conference (DDCRS), Bush House, King’s College London, 27 April 2024; at the 32nd Biennial Congress, the International Society of University Colon and Rectal Surgeons (ISUCRS) Seoul South Korea, 5–9 September 2024; at the Bio Century Grand Rounds, Four Season Hotel, Nashville, TN, USA, 9–11 September 2024; at the 124th American Society of Colon and Rectal Surgeons (ASCRS) Annual Scientific Congress, Seattle Convention Center, WA, USA, 3–6 June 2023; at the 10th Dilemmas & Debates in Colorectal Surgery Conference (DDCRS), Bush House, King’s College London, 12–14 January 2023; at the 122nd American Society of Colon and Rectal Surgeons (ASCRS) Annual Scientific Congress, Tampa Convention Center, FL, USA, 30 April–4 May 2022; at the 31st International Colorectal Research Summit (ICRS) and the Korean Society of Coloproctology (KSCP), 3–5 September 2021, Grand Walker Hill, Seoul, South Korea; at the European Society of Medicine’s Annual Congress (ESMED), Vienna, Austria, 27 September–1 October 2025; and at the 121st American Society of Colon and Rectal Surgeons (ASCRS) Annual Scientific Meeting, San Diego Convention Center, CA, USA, 24–28 April 2021.

## Data Availability

The data supporting the findings of this study are included in this article. Further inquiries can be directed to the corresponding author.
