# Correction: M’Koma, A.E. Secure Ileal Pouch–Anal Anastomosis for Histologic Indeterminate Colitis. *J. Clin. Med.* 2025, *14*, 8390

**DOI:** 10.3390/jcm15041519

**Published:** 2026-02-14

**Authors:** Amosy E. M’Koma

**Affiliations:** 1 Department of Biochemistry, Cancer Biology, Neuroscience and Pharmacology, Meharry Medical College School of Medicine, Nashville, TN 37208-3500, USA; amkoma@mmc.edu or amosy.e.mkoma@vumc.org; 2 Department of Pathology, Anatomy and Cell Biology, Meharry Medical College School of Medicine, Nashville General Hospital, Nashville, TN 37208-3599, USA; 3 Division of General Surgery, Section of Colon and Rectal Surgery, Vanderbilt University School of Medicine, Nashville, TN 37232-0260, USA; 4 The American Society of Colon and Rectal Surgeons (ASCRS), 2549 Waukegan Road, #210, Bannockburn, IL 600015, USA; 5 International Society of University Colon and Rectal Surgeons, 109 Partin Street, Chapel Hill, NC 27514, USA

## Error in Figure

In the original publication [1], there was a mistake in Figure 4A as published. Figure 4A,D are identical. The corrected Figure 4A appears below.

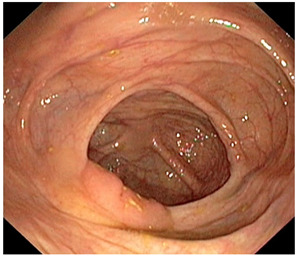



The author states that the scientific conclusions are unaffected. This correction was approved by the Academic Editor. The original publication has also been updated.
